# Higher Risk of Mortality and Virologic Failure in HIV-Infected Patients With High Viral Load at Antiretroviral Therapy Initiation: An Observational Cohort Study in Chongqing, China

**DOI:** 10.3389/fpubh.2022.800839

**Published:** 2022-02-03

**Authors:** Chao Zhou, Wei Zhang, Rongrong Lu, Lin Ouyang, Hui Xing, Yiming Shao, Guohui Wu, Yuhua Ruan

**Affiliations:** ^1^Chongqing Municipal Center for Disease Control and Prevention, Chongqing, China; ^2^State Key Laboratory of Infectious Disease Prevention and Control, National Center for AIDS/STD Control and Prevention, Chinese Center for Disease Control and Prevention, Collaborative Innovation Center for Diagnosis and Treatment of Infectious Diseases, Beijing, China

**Keywords:** HIV/AIDS, antiretroviral therapy, baseline, viral load, risk

## Abstract

**Background:**

Viral load (VL) is a strong predictor of human immunodeficiency virus (HIV) disease progression. The aim of this study was to evaluate the effect of high baseline VL on antiretroviral therapy (ART) outcomes among HIV-infected patients.

**Methods:**

This retrospective study observed HIV-infected patients who had baseline VL test at ART initiation between 2015 and 2019 in Chongqing, China. Cox proportional hazards regression and logistic regression models were used to evaluate the effects of baseline VL on Acquired immunodeficiency syndrome (AIDS)-related mortality and virologic failure, respectively.

**Results:**

The cohort included 7,176 HIV-infected patients, of whom 38.7% had a baseline VL ≥ 100,000 copies/mL. Of the patients who died during follow-up, 58.9% had a baseline VL ≥ 100,000 copies/mL. Compared with a baseline VL < 10,000 copies/mL, ART initiation at VL ≥ 100,000 copies/mL was significantly associated with the AIDS-related death (adjusted hazard ratio, AHR = 1.4) and virologic failure (adjusted odds ratio, AOR = 2.4). Compared with patients with a baseline VL < 10,000 copies/mL, patients on the recommended first-line regimen with a VL ≥ 100,000 copies/mL at ART initiaition had higher mortality rate (5.1 vs. 1.7 per 100 person-years), but there was no significant difference in the mortality accoding to the initial VL level among patients on second-line ART (2.8 vs. 2.7 per 100 person-years). ART initiation ≤ 30 days after HIV diagnosis was associated with a lower risk of AIDS-related death (AHR = 0.6).

**Conclusions:**

ART initiation with VL ≥ 100,000 copies/mL was associated with a significantly greater risk of mortality and virologic failure. Optimizing the ART regimen and initiating ART early may help to reduce mortality effectively among patients with a high baseline VL. VL testing for all HIV patients is recommended at HIV diagnosis or on ART initiation.

## Introduction

Currently, antiretroviral therapy (ART) is the most effective intervention to prevent new human immunodeficiency virus (HIV) infections and reduce the risk of Acquired immunodeficiency syndrome (AIDS)-associated mortality (1, 2). The World Health Organization recommends early initiation of ART for all newly diagnosed persons with HIV infection, regardless of the CD4 count. In China, the National Free Antiretroviral Treatment Program (NFATP) was initiated in 2003, and in line with the national AIDS control policy of “Four Frees and One Care”, has scaled-up provision of ART. By the end of 2019, more than 850,000 patients had received treatment nationwide, and 82.9% of patients on ART for at least 12 months had full virological suppression (3). With increased treatment coverage, the overall AIDS-associated mortality has steadily declined in China (4). Despite the rapid roll out of ART in China, HIV remains the leading cause of death among infectious diseases, and AIDS-associated mortality continues to be a public health concern of national importance (5, 6).

HIV viral load (VL) is significantly associated with the degree of active HIV replication, and a high VL predicts faster disease progression to AIDS and death (7). Monitoring of the VL level after ART initiation to assess effectiveness of treatment has been used increasingly in recent years (8, 9). However, baseline VL testing has remained under-utilized in resource-limited settings because of its high cost. Studies suggest that assessments of treatment outcomes should consider the CD4 counts, VL, and duration of ART (10). In the past, the baseline CD4 count was generally used to determine when to initiate ART, following a general consensus that early and immediate ART initiation after HIV diagnosis is most effective (11, 12), baseline CD4 testing is no longer recommended for deciding whether to initiate ART. More researchers have recommended a shift to VL monitoring before and after ART initiation in settings where HIV VL testing is available (13, 14).

It generally takes longer for HIV-infected patients to control viral replication and restore their immune system after ART initiation if they have a high VL. Patients with a high VL at the time of ART initiation are more likely to fail treatment or to develop low-level viremia (LLV) (15–17). In China, HIV infection is often diagnosed late, leading to a delay in presenting for treatment (18), so a considerable proportion of HIV patients initiate treatment with a high baseline VL.

However, there is no guidance on managing patients with a high baseline VL in China. Southwest China is the region with the highest burden of the HIV infection (19). Chongqing, which is located in the southwest China, currently plays an important role in the country's HIV prevention and treatment program (20). By the end of 2019, Chongqing reported ~50,000 surviving HIV patients and 14,000 AIDS-associated deaths. In order to understand the necessity of baseline VL testing, we conducted this retrospective observational cohort study to evaluate the effect of baseline VL on death and virologic failure after initiating treatment.

## Methods

### Study Design and Population

This retrospective observational cohort study of HIV patients initiatied on ART was conducted in Chongqing, China. Baseline VL was used to predict the treatment effects on patients' outcomes, including death and virologic failure. Data for individuals who were initiated ART between 2015 and 2019 were extracted from the NFATP database, which contained data on 35,056 individuals. All patients who had baseline VL test were selected. The eligibility criteria for inclusion in the analysis were; age ≥ 15 years when ART initiated, being newly initiated on treatment during the study period, and having follow-up records. A total of 7,176 (20.6%) individuals were included in the analysis. The cohort observations started on the date of ART initiation, with follow-up at 1, 2, and 3 months after ART initiation, and every 3 months thereafter, and terminated on December 31, 2020. All patients were followed up until death, loss to follow-up, or observation termination.

### Data Collection

ART data were extracted from the Chinese NFATP information system, a nationwide, real-time, reporting system, which is managed by the National Center for AIDS/STD Control and Prevention (NCAIDS) of the Chinese Center for Disease Control and Prevention (China CDC) (3). In China, all patients newly initiated on free ART and their follow-up information are required to be reported to the information system. Baseline data collected includes age, sex, marital status, transmission route, baseline CD4 counts, last ART regimen, duration from HIV diagnosis to ART, tuberculosis (TB) coinfection, hepatitis B virus coinfection, and baseline VL. The measure of death was AIDS-related mortality. The patient's death information was correlated with another Chinese HIV/AIDS comprehensive response information management system database (HIV case reporting database) by treatment code. A dropout event among surviving patients was defined as cessation of treatment or loss to follow-up for 90 days after missing a scheduled appointment. One-year virologic failure was defined as a VL ≥ 200 copies/mL at 12 (range 6–18) months after treatment initiation. Among patients who had more than one VL test, the VL result closest to the 12-month date was selected. The recommended first-line regimen was tenofovir, lamivudine, and efavirenz (TDF+3TC+EFV), and second-line regimens included lopinavir/ritonavir (LPV/r)-based, and abacavir (ABC)-based regimens, or other affordable options among self-paying patients. All records were anonymous and contained no private personal information. All patients signed an informed consent about ART at initiation of treatment, and their parents or guardians should also sign the informed consent if the patients were <16 years old. This study was approved by the Institutional Review Board of Chongqing CDC.

### Statistical Analysis

Death was treated as a time-dependent variable in the data analysis. The observed time was expressed in person-years (PYs). Chi-square tests were used to compare characteristics of HIV patients among baseline groups. Cox proportional hazards regression was used to assess the association between baseline VL and mortality. Logistic regression was used to evaluate the effect of baseline VL on virologic failure. In order to control for potential confounding, all baseline covariates were further entered the adjusted model based on univariate analyses. Based on baseline VL, all participants were categorized into three groups (< 10,000 copies/mL, 10,000–99,999 copies/mL, and ≥100,000 copies/mL). Other potential confounders of the model included age, sex, marital status, transmission route, baseline CD4 counts, last ART regimen, duration from HIV diagnosis to ART, TB coinfection, and hepatitis B virus coinfection. The adjusted hazard ratio (AHR) and adjusted odds ratio (AOR) were calculated using multivariable regression analysis. Two-sided *P*-values ≤ 0.05 were regarded as statistically significant. All statistical analyses were performed using IBM SPSS Statistics, Version 19.0 (IBM Corp., Armonk, NY, USA).

### Sensitivity Analysis of the Effect of Missing VL Data

A sensitivity analysis was conducted to assess the potential bias for individuals with missing VL data at initiation and at 12 months using Chi-square tests. We compared the baseline characteristics of HIV patients according to whether they received a baseline VL test and a VL test at 12 months (Supplementary Tables 1, 2). The results showed that the baseline characteristics of HIV patients with and without a baseline VL result were slightly different. Baseline VL testing was not freely available in all facilities, but as many patients as possible were included in the analysis.

## Results

### Demographic Characteristics of HIV-Infected Patients

A total of 7,176 HIV-infected patients met the inclusion criteria. The patient characteristics are shown in Table 1. Most individuals were aged between 15–49 years (62.1%). 78.7% were males, and 51.0% were married. The main HIV infection route was heterosexual contact (63.0%), and the most common first-line ART regimen was TDF+3TC+EFV (79.1%). Approximately half of the individuals (50.1%) had CD4 counts <200 cells/mm^3^ at ART initiation, and most individuals (62.9%) started ART within 30 days after diagnosis. A total of 9.4% of individuals had TB coinfection, and 9.0% had hepatitis B virus coinfection. The percentage of patients with baseline VL results < 10,000 copies/mL, 10,000–99,999 copies/mL, and ≥100,000 copies/mL was 21.0% (*n* = 1,503), 40.3% (*n* = 2,893) and 38.7% (*n* = 2,780), respectively. Among patients with a baseline VL < 10,000 copies/mL, 28.5% had a baseline CD4 count < 200 cells/mm^3^, while among patients with a baseline VL ≥ 100,000 copies/mL, 72.7% had a baseline CD4 count <200 cells/mm^3^.

**Table 1:** Characteristics of HIV patients initiating ART between 2015 and 2019 in Chongqing, China.

**Variables**	**Entire study cohort, ***N*** (%)**	**Baseline viral load (copies/mL),** ***N*** **(%)**			
		**10,000**	**10,000–99,999**	**≥100,000**	***P*** -value*
Total	7,176 (100)	1,503 (100)	2,893 (100)	2,780 (100)	
Age(years)					<0.001
xs 15–49	4,456 (62.1)	1,011 (67.3)	1,925 (66.5)	1,520 (54.7)	
≥50	2,720 (37.9)	492 (32.7)	968 (33.5)	1,260 (45.3)	
Sex					0.001
Male	5,646 (78.7)	1,132 (75.3)	2,271 (78.5)	2,243 (80.7)	
Female	1,530 (21.3)	371 (24.7)	622 (21.5)	537 (19.3)	
Marital status					<0.001
Single	2,405 (33.5)	520 (34.6)	1,132 (39.1)	753 (27.1)	
Married	3,659 (51.0)	756 (50.3)	1,365 (47.2)	1,538 (55.3)	
Divorced and others	1,112 (15.5)	227 (15.1)	396 (13.7)	489 (17.6)	
Transmission route					<0.001
Heterosexual contact	4,526 (63.0)	912 (60.7)	1,670 (57.7)	1,944 (69.9)	
Homosexual contact	2,267 (31.6)	487 (32.4)	1,100 (38.0)	680 (24.5)	
Injection drug use	55 (0.8)	18(1.2)	22 (0.8)	15 (0.5)	
Unknown	328 (4.6)	86 (5.7)	101 (3.5)	141 (5.1)	
Baseline CD4 counts (cells/mm^3^)					<0.001
<200	3,598 (50.1)	428 (28.5)	1,148 (39.7)	2,022 (72.7)	
200–499	3,034 (42.3)	868 (57.8)	1,513 (52.3)	653 (23.5)	
≥500	428 (6.0)	173 (11.5)	188 (6.5)	67 (2.4)	
Missing	116 (1.6)	34 (2.2)	44 (1.5)	38 (1.4)	
Last ART regimen					<0.001
Recommended first-line regimen	5,678 (79.1)	1,269 (84.4)	2,399 (82.9)	2,010 (72.3)	
Alternative first-line regimens	202 (2.8)	50 (3.3)	61 (2.1)	91 (3.3)	
Second-line regimens	1,296 (18.1)	184 (12.3)	433 (15.0)	679 (24.4)	
Duration from HIV diagnosis to ART (days)				<0.001
>30	2,664 (37.1)	660 (43.9)	1,106 (38.2)	898 (32.3)	
≤ 30	4,512 (62.9)	843 (56.1)	1,787 (61.8)	1,882 (67.7)	
TB coinfection					<0.001
No	6,464 (90.1)	1,429 (95.1)	2,738 (94.6)	2,297 (82.6)	
Yes	679 (9.4)	60 (4.0)	149 (5.2)	470 (16.9)	
Missing	33 (0.5)	14 (0.9)	6 (0.2)	13 (0.5)	
Hepatitis B virus coinfection					0.048
No	5,179 (72.2)	1,044 (69.5)	2,102 (72.7)	2,033 (73.1)	
Yes	645 (9.0)	137 (9.1)	269 (9.3)	239 (8.6)	
Missing	1,352 (18.8)	322 (21.4)	522 (18.0)	508 (18.3)	

*Recommended first-line regimen: tenofovir, lamivudine, and efavirenz (TDF+3TC+EFV); Alternative first-line regimens: zidovudine, lamivudine, and nevirapine (AZT+3TC+NVP), zidovudine, lamivudine, and efavirenz (AZT+3TC+EFV), tenofovir, lamivudine, and nevirapine (TDF+3TC+NVP); Second-line regimens: regimens including lopinavir/ritonavir (LPV/r), abacavir (ABC), or other affordable self-paying therapeutic options; TB, tuberculosis*;

* *Chi-square tests*.

### Effect of Baseline VL on Death

A total of 557 patients experienced an AIDS-related death after initiating ART, accounting for 7.8% of the cohort. The overall AIDS-related mortality rate was 2.9 per 100 PYs (Table 2). The proportion of deaths according to baseline VL < 10,000 copies/mL, 10,000–99,999 copies/mL, and ≥100,000 copies/mL was 14.9% (*n* = 83), 26.2% (*n* = 146) and 58.9% (*n* = 328), respectively. ART initiation at VL ≥ 100,000 copies/mL was associated with a significantly higher mortality (AHR = 1.4, 95% CI: 1.1–1.8). Other factors associated with a significantly increased risk of death after initiating ART included age ≥50 years old (AHR = 2.4, 95% CI: 2.0–3.0), use of alternative first-line regimens (AHR = 2.3, 95% CI: 1.7–3.3), TB coinfection (AHR = 1.8, 95% CI: 1.4–2.2), hepatitis B virus coinfection (AHR = 1.3, 95% CI: 1.0–1.7). Factors associated with a significantly decreased risk of death included use of second-line regimens (AHR = 0.7, 95% CI: 0.6–0.9), female sex (AHR = 0.6, 95% CI: 0.5–0.7), homosexual contact (AHR = 0.6, 95% CI: 0.4–0.8), ART initiation ≤ 30 days after HIV diagnosis (AHR = 0.6, 95% CI: 0.5–0.8), and a baseline CD4 count of 200–499 cells/mm^3^ (AHR = 0.4, 95% CI: 0.4–0.6) or ≥500 cells/mm^3^ (AHR = 0.3, 95% CI: 0.2–0.6).

**Table 2:** Effects of baseline viral load on death among HIV patients initiating ART in Chongqing, China, 2015–2019.

**Variables**	**Number**	**Deaths** ***N*** **(%)**	**Observed time (PYs)**	**Mortality rate per 100 PYs**	**AHR** **(95% CI)**	***P*** **-value**
Total	7,176	557 (100)	19,234.8	2.9 (2.7, 3.1)		
**Age (years)**						
18–49	4,456	208 (37.3)	12,899.1	1.6 (1.4, 1.8)	Ref	
≥50	2,720	349 (62.7)	6,335.7	5.5 (4.9, 6.1)	2.4 (2.0, 3.0)	<0.001
**Sex**						
Male	5,646	468 (84.0)	15,342.1	3.1 (2.8, 3.3)	Ref	
Female	1,530	89 (16.0)	3,892.7	2.3 (1.8, 2.8)	0.6 (0.5, 0.7)	<0.001
**Marital status**						
Single	2,405	106 (19.0)	7,130.2	1.5 (1.2, 1.8)	Ref	
Married	3,659	326 (58.5)	9,286.7	3.5 (3.1, 3.9)	0.9 (0.7, 1.2)	0.672
Divorced and others	1,112	125 (22.5)	2,817.9	4.4 (3.7, 5.2)	1.2 (0.9, 1.6)	0.320
**Transmission route**						
Heterosexual contact	4,526	432 (77.6)	11,266.4	3.8 (3.5, 4.2)	Ref	
Homosexual contact	2,267	82 (14.7)	6,924.2	1.2 (0.9, 1.4)	0.6 (0.4, 0.8)	<0.001
Injection drug use	55	3 (0.5)	148.8	2.0 (0.0, 4.3)	0.6 (0.2, 1.9)	0.377
Unknown	328	40 (7.2)	895.4	4.5 (3.1, 5.8)	1.2 (0.8, 1.6)	0.306
**Baseline CD4 counts (cells/mm** ^**3**^ **)**						
<200	3,598	423 (75.9)	9,147.5	4.6 (4.2, 5.1)	Ref	
200–499	3,034	116 (20.8)	8,607.2	1.3 (1.1, 1.6)	0.4 (0.4, 0.6)	<0.001
≥500	428	11 (2.0)	1,217.8	0.9 (0.4, 1.4)	0.3 (0.2, 0.6)	<0.001
Missing	116	7 (1.3)	262.3	2.7 (0.7, 4.6)	0.6 (0.3, 1.3)	0.210
**Last ART regimen**						
Recommended first-line regimen	5,678	432 (77.6)	15,480.1	2.8 (2.5, 3.1)	Ref	
Alternative first-line regimens	202	39 (7.0)	512.7	7.6 (5.3, 9.9)	2.3 (1.7, 3.3)	<0.001
Second-line regimens	1,296	86 (15.4)	3,242.1	2.7 (2.1, 3.2)	0.7 (0.6, 0.9)	0.016
**Duration from HIV diagnosis to ART (days)**						
>30	2,664	249 (44.7)	7,626.3	3.3 (2.9, 3.7)	Ref	
≤ 30	4,512	308 (55.3)	11,608.5	2.7 (2.4, 2.9)	0.6 (0.5, 0.8)	<0.001
**TB coinfection**						
No	6,464	427 (76.7)	17,399.5	2.5 (2.2, 2.7)	Ref	
Yes	679	123 (22.1)	1,749.5	7.0 (5.8, 8.2)	1.8 (1.4, 2.2)	<0.001
Missing	33	7 (1.2)	85.8	8.2 (2.2, 14.0)	2.0 (0.9, 4.2)	0.081
**Hepatitis B virus coinfection**						
No	5,179	395 (70.9)	14,055.7	2.8 (2.5, 3.1)	Ref	
Yes	645	65 (11.7)	1,702.8	3.8 (2.9, 4.7)	1.3 (1.0, 1.7)	0.036
Missing	1,352	97 (17.4)	3,476.3	2.8 (2.2, 3.3)	1.0 (0.8, 1.3)	0.983
**Baseline viral load (copies/mL)**						
< 10,000	1,503	83 (14.9)	4,298.5	1.9 (1.5, 2.3)	Ref	
10,000–99,999	2,893	146 (26.2)	8,088.7	1.8 (1.5, 2.1)	0.9 (0.7, 1.2)	0.362
≥100,000	2,780	328 (58.9)	6,847.8	4.8 (4.3, 5.3)	1.4 (1.1, 1.8)	0.006

*Recommended first-line regimen: tenofovir, lamivudine, and efavirenz (TDF+3TC+EFV); Alternative first-line regimens: zidovudine, lamivudine, and nevirapine (AZT+3TC+NVP), zidovudine, lamivudine, and efavirenz (AZT+3TC+EFV), tenofovir, lamivudine, and nevirapine (TDF+3TC+NVP); Second-line regimens: regimens including lopinavir/ritonavir (LPV/r), abacavir (ABC), or other affordable self-paying therapeutic options; TB, tuberculosis; Ref, reference; PYs, person years; AHR, adjusted hazard ratio*.

### Effect of Baseline VL on Virologic Failure

Of the 5,440 patients who had VL tested at 12 months after treatment initiation, 284 patients (5.2%) exhibited virologic failure (Table 3). Among the patients with virologic failure, 60.6% (*n* = 172) had a baseline VL ≥ 100,000 copies/mL. The virologic failure rate of patients with a baseline VL < 10,000 copies/mL, 10,000–99,999 copies/mL, and ≥100,000 copies/mL was 2.8, 3.5, and 8.5%, respectively. The adjusted logistic regression model showed that a baseline VL ≥ 100,000 copies/mL was associated with an increased risk of virologic failure, compared with a baseline VL < 10,000 copies/mL (AOR = 2.4, 95% CI: 1.6–3.7).

**Table 3:** Effects of baseline viral load on virologic failure at 12 months among HIV patients initiating ART in Chongqing, China, 2015–2019.

**Variables**	**Number**	**Virologic failure** ***N*** **(%)**	**Virologic failure rate % (95% CI)**	**AOR** **(95% CI)**	***P*** **-value**
Overall	5440	284 (100)	5.2 (4.6, 5.8)		
**Baseline viral load (copies/mL)**					
< 10,000	1,134	32 (11.3)	2.8 (1.9, 3.8)	Ref	
10,000–99,999	2,289	80 (28.1)	3.5 (2.7, 4.2)	1.2 (0.8, 1.9)	0.366
≥100,000	2,017	172 (60.6)	8.5 (7.3, 9.7)	2.4 (1.6, 3.7)	<0.001

*AOR, adjusted odds ratio; virologic failure: VL ≥ 200 copies/ml; covariates of the adjusted model included: age, sex, marital status, transmission route, baseline CD4 counts, last ART regimen, duration from HIV diagnosis to ART, TB coinfection, and Hepatitis B virus coinfection*.

### Effect of Baseline VL on Mortality According to the ART Regimen

A further analysis of mortality according to baseline VL, stratified by the last ART regimen is shown in Table 4. Among patients on the recommended first-line regimen, patients with a VL ≥ 100,000 copies/mL at ART initiation had a higher mortality rate than those with a VL < 10,000 copies/mL (5.1 vs. 1.7 per 100 PYs), but there was no significant difference in the group of patients treated with second-line regimens (2.8 vs. 2.7 per 100 PYs).

**Table 4:** Effects of baseline viral load on death stratified by last ART regimen among HIV patients initiating ART in Chongqing, China, 2015–2019.

**Variables**	**Recommended first-line regimen**				**Second-line regimens**			
	**Deaths**	**Mortality rate per 100 PYs**	**AHR** **(95% CI)**	***P*** **-value**	**Deaths**	**Mortality rate per 100 PYs**	**AHR** **(95% CI)**	***P*** **-value**
Overall	432	2.8 (2.5, 3.1)			86	2.7 (2.1, 3.2)		
**Baseline viral load (copies/mL)**								
< 10,000	62	1.7 (1.3, 2.1)	Ref		14	2.7 (1.3, 4.1)	Ref	
10,000–99,999	111	1.6 (1.3, 1.9)	0.9 (0.6, 1.2)	0.339	56	2.4 (1.5, 3.3)	0.8 (0.4, 1.6)	0.548
≥100,000	259	5.1 (4.5, 5.8)	1.6 (1.2, 2.2)	0.001	16	2.8 (2.0, 3.7)	0.6 (0.3, 1.1)	0.112

*PYs, person years; AHR, adjusted hazard ratio; covariates of the adjusted model included: age, sex, marital status, transmission route, baseline CD4 counts, duration from HIV diagnosis to ART, TB coinfection, and Hepatitis B virus coinfection; Recommended first-line regimen: tenofovir, lamivudine, and efavirenz (TDF+3TC+EFV); Second-line regimens: regimens including lopinavir/ritonavir (LPV/r), abacavir (ABC), or other affordable self-paying therapeutic options*.

## Discussion

In this study, a considerable proportion (38.7%) of HIV-infected patients had a high VL (≥100,000 copies/mL) on starting ART. This result is similar to that of an international cohort of European and Australian patients (36.8%) (21). The overall AIDS-related mortality rate in the current study (2.9 per 100 PYs) was consistent with that of a previous study conducted in Chongqing, China (3.0 per 100 PYs) (22), and was similar to a study reported in southwest China's Guangxi Zhuang Autonomous Region in 2017 (2.63 per 100 PYs) (23), so the data of this study were representative of patients with HIV in southwest China.

Our study showed that high baseline VL was one of the factors associated with ART mortality. Approximately 40% of HIV-infected individuals had a baseline VL ≥ 100,000 copies/mL, while ~60% of AIDS-related deaths after starting ART occurred among patients with a baseline VL ≥ 100,000 copies/mL. The adjusted Cox proportional hazards regression model showed that a baseline VL ≥ 100,000 copies/mL increased the hazard of mortality by 40% compared with a baseline VL < 10,000 copies/mL. We suggest strengthening baseline VL measurement among all HIV patients. Currently routine baseline VL testing is not recommended for all HIV patients in China. Most county-level ART clinics have the resources needed to measure VL. Efforts should be made to increase the availability of low-cost equipment for measuring HIV viral load.

Virologic failure and low baseline CD4 counts may have accounted for the increased risk of death among patients with a high baseline VL. It generally takes several months after initiation of ART to achieve VL suppression (24). However, patients with high baseline VL might have a larger HIV reservoir, and may take them longer to achieve viral suppression and immune reconstitution (25). In this study, patients with a high baseline VL had a greater risk of virologic failure at 12 months on ART (AOR = 2.4). Another study conducted in China found that patients with a baseline VL > 100,000 copies/mL were 3.2 times more likely to experience virologic failure (26), and a study on youth with HIV infection found that a low baseline VL was a predictor of viral suppression (AHR = 1.56) (27). Additionally, a high baseline VL can continue to damage the immune system and increase the possibility of opportunistic infections and AIDS-related mortality (28, 29). A study on TB-HIV patients found that patients with higher baseline VL were more likely to experience virologic failure (AOR=1.5) (30). Our study also showed that among patients with baseline VL ≥ 100,000 copies/mL, >70% had a baseline CD4 count <200 cells/mm^3^, and 17% had TB coinfection. Therefore, HIV patients with high baseline VL are more likely to have poorer treatment outcomes and more disease progression.

These results suggest that optimizing the ART regimen and initiating ART early may help to limit mortality among patients with high baseline VL. A study in Henan region of China showed that long-term second-line ART was effective (31). Another study in Myanmar showed that 66.5% patients with virologic failure were switched to a second-line ART regimen within 6 months after confirmed virologic failure (32). We found that second-line ART was useful for reducing the overall cohort mortality rate, and that among patients on second-line ART regimens, the mortality rates did not differ significantly according to the baseline VL. The proportion of patients that switched to second-line ART was higher in those with a high baseline VL than in those with a baseline VL < 10,000 copies/mL. The reason may be that patients with a high VL may have been started on second-line ART directly or switched to second-line ART after first-line ART failure. During the past 20 years, China has made a great effort to increase access to ART regimens and has continually sought to evaluate and optimize these regimens (33). Patients with a high baseline VL and virologic failure should be switched to a second-line regimen as soon as possible, even without drug resistance results. Additionally, consistent with several other studies (22), the results of this study suggest that early treatment ( ≤ 30 days after HIV diagnosis) may be associated with reduced mortality. Immediate ART initiation has been included in the Chinese national guidelines, but one third of patients still initiated treatment above 30 days after diagnosis. Moreover, there is an increased risk of HIV transmission to partners when patients have a high VL for a longer time. For reducing HIV transmission, it is more valuable to provide VL testing at HIV diagnosis by employing VL-triggered early treatment.

Our study has some limitations. First, a notable proportion of individuals were excluded from the study because they did not have a baseline VL, which may have biased the results. Many patients did not have a baseline VL result because baseline VL testing is not recommended for all patients in China. Second, records of virologic failure only included individuals tested for VL during the 6–18 months following ART initiation; therefore, some patients without VL results during this time window were excluded from the analysis. Third, the number of deaths might have been underestimated due to unascertained deaths being reported as dropouts.

## Conclusion

Our study indicated that a considerable proportion of patients had high baseline VL, and that ART initiation with high VL was significantly associated with a greater risk of mortality and virologic failure. Optimizing the ART regimen and initiating ART early may help to reduce mortality among patients with a high baseline VL. All HIV patients should have a baseline VL test at ART initiation. In order to reduce HIV transmission, ideally a VL test should be provided at the time of HIV diagnosis.

## Data Availability Statement

The original contributions presented in the study are included in the article/Supplementary Material, further inquiries can be directed to the corresponding author/s.

## Ethics Statement

The studies involving human participants were reviewed and approved by Institutional Review Board of Chongqing CDC. Written informed consent to participate in this study was provided by the participants' legal guardian/next of kin.

## Author Contributions

CZ, GW, and YR drafted the manuscript. WZ, RL, and LO collected data. HX and YS guided the article design. All authors read and approved the final manuscript.

## Funding

This work was supported by the Research Project of Chongqing Municipal Science and Technology Bureau (cstc2019jscx-msxmX0225), Chongqing medical scientific research project of Chongqing Health Commission and Science and Technology Bureau (2022GDRC017), National Natural Science Foundation of China (11971479), and Chinese State Key Laboratory of Infectious Disease Prevention and Control.

## Conflict of Interest

The authors declare that the research was conducted in the absence of any commercial or financial relationships that could be construed as a potential conflict of interest.

## Publisher's Note

All claims expressed in this article are solely those of the authors and do not necessarily represent those of their affiliated organizations, or those of the publisher, the editors and the reviewers. Any product that may be evaluated in this article, or claim that may be made by its manufacturer, is not guaranteed or endorsed by the publisher.

## References

[1] Vella S Schwartländer B Sow SP Eholie SP Murphy RL. The history of antiretroviral therapy and of its implementation in resource-limited areas of the world. AIDS. (2012) 26:1231–41. 10.1097/QAD.0b013e32835521a3 22706009

[2] Cohen MS Chen YQ McCauley M Gamble T Hosseinipour MC Kumarasamy N. Prevention of HIV-1 infection with early antiretroviral therapy. N Engl J Med. (2011) 365:493–505. 10.1056/NEJMoa1105243 21767103 PMC3200068

[3] Zhao Y Han MJ Gan XM Ma Y Zhao DC. Characteristics and viral suppression among people living with HIV from the national free antiretroviral therapy programme, 2019. HIV Med. (2020) 21:701–7. 10.1111/hiv.13020 33369034

[4] Zhang F Dou Z Ma Y Zhang Y Zhao Y Zhao D. Effect of earlier initiation of antiretroviral treatment and increased treatment coverage on HIV-related mortality in China: a national observational cohort study. Lancet Infect Dis. (2011) 11:516–24. 10.1016/S1473-309970097-4 21600849

[5] Dong Y Wang L Burgner DP Miller JE Song Y Ren X. Infectious diseases in children and adolescents in China: analysis of national surveillance data from 2008 to 2017. BMJ. (2020) 369:m1043. 10.1136/bmj.m1043 32241761 PMC7114954

[6] Wang J Yuan T Ling X Li Q Tang X Cai W. Critical appraisal and external validation of a prognostic model for survival of people living with HIV/AIDS who underwent antiretroviral therapy. Diagn Progn Res. (2020) 4:19. 10.1186/s41512-020-00088-x 33292789 PMC7687783

[7] Eller MA Opollo MS Liu M Redd AD Eller LA Kityo C. HIV Type 1 disease progression to AIDS and death in a rural Ugandan cohort is primarily dependent on viral load despite variable subtype and T-cell immune activation levels. J Infect Dis. (2015) 211:1574–84. 10.1093/infdis/jiu646 25404522 PMC4425824

[8] Kadima J Patterson E Mburu M Blat C Nyanduko M Bukusi EA. Adoption of routine virologic testing and predictors of virologic failure among HIV-infected children on antiretroviral treatment in western Kenya. PLoS ONE. (2018) 13:e0200242. 10.1371/journal.pone.0200242 30412576 PMC6226151

[9] Brijkumar J Johnson BA Zhao Y Edwards J Moodley P Pathan K. A packaged intervention to improve viral load monitoring within a deeply rural health district of South Africa. BMC Infect Dis. (2020) 20:836. 10.1186/s12879-020-05576-5 33176715 PMC7659110

[10] Brennan AT Maskew M Sanne I Fox MP. The interplay between CD4 cell count, viral load suppression and duration of antiretroviral therapy on mortality in a resource-limited setting. Trop Med Int Health. (2013) 18:619–31. 10.1111/tmi.12079 23419157 PMC3625450

[11] Fatti G Grimwood A Nachega JB Nelson JA LaSorda K van Zyl G. Better virological outcomes among people living with human immunodeficiency virus (HIV) initiating early antiretroviral treatment (CD4 counts ≥500 cells/μL) in the HIV Prevention Trials Network 071 (PopART) Trial in South Africa. Clin Infect Dis. (2020) 70:395–403. 10.1093/cid/ciz214 30877753 PMC7768744

[12] Zhao Y McGoogan JM Wu Z. The benefits of immediate ART. J Int Assoc Provid AIDS Care. (2019) 18:2325958219831714. 10.1177/2325958219831714 30832530 PMC6748486

[13] Shoko C Chikobvu D. A superiority of viral load over CD4 cell count when predicting mortality in HIV patients on therapy. BMC Infect Dis. (2019) 19:169. 10.1186/s12879-019-3781-1 30770728 PMC6377778

[14] Ford N Meintjes G Vitoria M Greene G Chiller T. The evolving role of CD4 cell counts in HIV care. Curr Opin HIV AIDS. (2017) 12:123–8. 10.1097/COH.0000000000000348 28059957

[15] Joao EC Gouvêa MI Menezes JA Sidi LC Cruz ML Berardo PT. Factors associated with viral load suppression in HIV-infected pregnant women in Rio de Janeiro, Brazil. Int J STD AIDS. (2012) 23:44–7. 10.1258/ijsa.2011.010545 22362687

[16] Chen S Han Y Song XJ Li YL Zhu T Lu HZ. Very high baseline HIV viremia impairs efficacy of non-nucleoside reverse transcriptase inhibitor-based ART: a long-term observation in treatment-naïve patients. Infect Dis Poverty. (2020) 9:75. 10.1186/s40249-020-00700-8 32571409 PMC7310120

[17] Zhang T Ding H An M Wang X Tian W Zhao B. Factors associated with high-risk low-level viremia leading to virologic failure: 16-year retrospective study of a Chinese antiretroviral therapy cohort. BMC Infect Dis. (2020) 20:147. 10.1186/s12879-020-4837-y 32066392 PMC7026956

[18] Tang H Mao Y Tang W Han J Xu J Li J. “Late for testing, early for antiretroviral therapy, less likely to die”: Results from a large HIV cohort study in China, 2006-2014. BMC Infect Dis. (2018) 18:272. 10.1186/s12879-018-3158-x 29895275 PMC5998580

[19] Wu Z Chen J Scott SR McGoogan JM. History of the HIV epidemic in China. Curr HIV/AIDS Rep. (2019) 16:458–66. 10.1007/s11904-019-00471-4 31773405 PMC7088640

[20] Wu G Zhou C Zhang X Zhang W Lu R Ouyang L. Higher risks of virologic failure and all-cause deaths among older people living with HIV in Chongqing, China. AIDS Res Hum Retroviruses. (2019) 35:1095–102. 10.1089/AID.2019.0096 31544479 PMC6862950

[21] Mocroft A Neesgard B Zangerle R Rieger A Castagna A Spagnuolo V. Treatment outcomes of integrase inhibitors, boosted protease inhibitors and nonnucleoside reverse transcriptase inhibitors in antiretroviral-naïve persons starting treatment. HIV Med. (2020) 21:599–606. 10.1111/hiv.12888 32588958

[22] Zhou C Zhang W Lu RR Ouyang L Xing H Shao YM. Benefits of early and immediate initiation of antiretroviral therapy among HIV patients in Chongqing, China. Biomed Environ Sci. (2020) 33:282–5. 10.3967/bes2020.039 32438967

[23] Tang Z Pan SW Ruan Y Liu X Su J Zhu Q. Effects of high CD4 cell counts on death and attrition among HIV patients receiving antiretroviral treatment: an observational cohort study. Sci Rep. (2017) 7:3129. 10.1038/s41598-017-03384-7 28600549 PMC5466653

[24] Ali JH Yirtaw TG. Time to viral load suppression and its associated factors in cohort of patients taking antiretroviral treatment in East Shewa Zone, Oromiya, Ethiopia, 2018. BMC Infect Dis. (2019) 19:1084. 10.1186/s12879-019-4702-z 31881859 PMC6935054

[25] Hussen S Mama M Mekonnen B Yihune M Shegaze M Boti N. Predictors of time to viral load suppression of adult PLWHIV on ART in Arba Minch General Hospital: a follow up study. Ethiop J Health Sci. (2019) 29:751–8. 10.4314/ejhs.v29i6.12 31741646 PMC6842721

[26] Wang X Yang L Li H Zuo L Liang S Liu W. Factors associated with HIV virologic failure among patients on HAART for one year at three sentinel surveillance sites in China. Curr HIV Res. (2011) 9:103–11. 10.2174/157016211795569122 21361864

[27] Kapogiannis BG Koenig LJ Xu J Mayer KH Loeb J Greenberg L. The HIV continuum of care for adolescents and young adults attending 13 urban US HIV care centers of the NICHD-ATN-CDC-HRSA SMILE Collaborative. J Acquir Immune Defic Syndr. (2020) 84:92–100. 10.1097/QAI.0000000000002308 32267659 PMC7147723

[28] Bartlett AW Mohamed TJ Sudjaritruk T Kurniati N Nallusamy R Hansudewechakul R. Disease- and treatment-related morbidity in adolescents with perinatal HIV infection in Asia. Pediatr Infect Dis J. (2019) 38:287–92. 10.1097/INF.0000000000002208 30281549 PMC6375771

[29] McGuire JL Gill AJ Douglas SD Kolson DL CNS HIV Anti-Retroviral Therapy Effects Research (CHARTER) group. Central and peripheral markers of neurodegeneration and monocyte activation in HIV-associated neurocognitive disorders. J Neurovirol. (2015) 21:439–48. 10.1007/s13365-015-0333-3 25776526 PMC4511078

[30] Demitto FO Schmaltz CAS Sant'Anna FM Arriaga MB Andrade BB Rolla VC. Predictors of early mortality and effectiveness of antiretroviral therapy in TB-HIV patients from Brazil. PLoS ONE. (2019) 14:e0217014. 10.1371/journal.pone.0217014 31170171 PMC6553696

[31] Chen J Zhang M Shang M Yang W Wang Z Shang H. Research on the treatment effects and drug resistances of long-term second-line antiretroviral therapy among HIV-infected patients from Henan Province in China. BMC Infect Dis. (2018) 18:571. 10.1186/s12879-018-3489-7 30442114 PMC6238347

[32] Mesic A Spina A Mar HT Thit P Decroo T Lenglet A. Predictors of virological failure among people living with HIV receiving first line antiretroviral treatment in Myanmar: Retrospective cohort analysis. AIDS Res Ther. (2021) 18:16. 10.1186/s12981-021-00336-0 33882962 PMC8059266

[33] Cao W Hsieh E Li T. Optimizing treatment for adults with HIV/AIDS in China: successes over two decades and remaining challenges. Curr HIV/AIDS Rep. (2020) 17:26–34. 10.1007/s11904-019-00478-x 31939111 PMC6989417

