# Supplementary material for: Higher Risk of Mortality and Virologic Failure in HIV-Infected Patients With High Viral Load at Antiretroviral Therapy Initiation: An Observational Cohort Study in Chongqing, China
Source: Front Public Health. 2022 Feb 3;10:800839. doi: 10.3389/fpubh.2022.800839 (PMC8851314; doi:10.3389/fpubh.2022.800839)
Supplement: Supplementary file 1 [file Data_Sheet_1.doc]

**APPENDIX**

**Supplementary Table 1.** Characteristics of HIV patients initiating ART by receipt of baseline VL test (n=35,056)

| Variables | Total (n=35,056) | (%) | Baseline VL result available (n=7,176) | (%) | Baseline VL result not available (n=27,880) | (%) | *P* value^*^ |
| --- | --- | --- | --- | --- | --- | --- | --- |
| Age(years) |  |  |  |  |  |  | <0.001 |
| 15–49 | 18543 | 52.9 | 4456 | 62.1 | 14087 | 50.5 |  |
| ≥50 | 16513 | 47.1 | 2720 | 37.9 | 13793 | 49.5 |  |
| Sex |  |  |  |  |  |  | <0.001 |
| Male | 26288 | 75.0 | 5646 | 78.7 | 20642 | 74.0 |  |
| Female | 8768 | 25.0 | 1530 | 21.3 | 7238 | 26.0 |  |
| Marital status |  |  |  |  |  |  | <0.001 |
| Single | 8511 | 24.2 | 2405 | 33.5 | 6106 | 21.9 |  |
| Married | 20247 | 57.8 | 3659 | 51.0 | 16588 | 59.5 |  |
| Divorced and others | 6298 | 18.0 | 1112 | 15.5 | 5186 | 18.6 |  |
| Transmission route |  |  |  |  |  |  | <0.001 |
| Heterosexual contact | 25765 | 73.5 | 4526 | 63.0 | 21239 | 76.1 |  |
| Homosexual contact | 6411 | 18.2 | 2267 | 31.6 | 4144 | 14.9 |  |
| Injection drug use | 965 | 2.8 | 55 | 0.8 | 910 | 3.3 |  |
| Unknown | 1915 | 5.5 | 328 | 4.6 | 1587 | 5.7 |  |
| Baseline CD4 counts (cells/mm^3^) |  |  |  |  |  |  | <0.001 |
| <200 | 12840 | 36.6 | 3598 | 50.1 | 9242 | 33.1 |  |
| 200–499 | 15260 | 43.5 | 3034 | 42.3 | 12226 | 43.9 |  |
| ≥500 | 2260 | 6.4 | 428 | 6.0 | 1832 | 6.6 |  |
| Missing | 4696 | 13.5 | 116 | 1.6 | 4580 | 16.4 |  |
| Last ART regimen |  |  |  |  |  |  | <0.001 |
| Recommended first-line regimen | 29310 | 83.6 | 5678 | 79.1 | 23632 | 84.8 |  |
| Alternative first-line regimens | 2628 | 7.5 | 202 | 2.8 | 2426 | 8.7 |  |
| Second-line regimens | 3118 | 8.9 | 1296 | 18.1 | 1822 | 6.5 |  |
| Duration from HIV diagnosis to ART (days) |  |  |  |  |  |  | <0.001 |
| >30 | 20171 | 57.5 | 2664 | 37.1 | 12221 | 43.8 |  |
| ≤30 | 14885 | 42.5 | 4512 | 62.9 | 15659 | 56.2 |  |
| TB coinfection |  |  |  |  |  |  | <0.001 |
| No | 32778 | 93.5 | 6464 | 90.1 | 26314 | 94.4 |  |
| Yes | 1385 | 4.0 | 679 | 9.4 | 706 | 2.5 |  |
| Missing | 893 | 2.5 | 33 | 0.5 | 860 | 3.1 |  |
| Hepatitis B virus coinfection |  |  |  |  |  |  | <0.001 |
| No | 20738 | 59.1 | 5179 | 72.2 | 15559 | 55.8 |  |
| Yes | 2267 | 6.5 | 645 | 9.0 | 1622 | 5.8 |  |
| Missing | 12051 | 34.4 | 1352 | 18.8 | 10699 | 38.4 |  |
| Deaths |  |  |  |  |  |  | <0.001 |
| Yes | 3913 | 11.1 | 557 | 7.8 | 3356 | 12.0 |  |
| No | 31143 | 88.9 | 6619 | 92.2 | 24524 | 88.0 |  |

***Note.*** VL, viral load; Recommended first-line regimen: tenofovir, lamivudine, and efavirenz (TDF+3TC+EFV); Alternative first-line regimens: zidovudine, lamivudine, and nevirapine (AZT+3TC+NVP), zidovudine, lamivudine, and efavirenz (AZT+3TC+ EFV), tenofovir, lamivudine, and nevirapine (TDF+3TC+NVP); Second-line regimens: regimens including lopinavir/ritonavir (LPV/r), abacavir (ABC), or other affordable self-paying therapeutic options; TB, tuberculosis; ^*^Chi-square test.

**Supplementary Table 2.** Characteristics of HIV patients with baseline VL according to one-year VL result (n=7,176)

| Variables | Total (n=7,176) | (%) | VL result available (n=5,440) | (%) | VL result not available (n=1,736) | (%) | *P* value^*^ |
| --- | --- | --- | --- | --- | --- | --- | --- |
| Age(years) |  |  |  |  |  |  | <0.001 |
| 15–49 | 4456 | 62.1 | 3485 | 64.1 | 971 | 55.9 |  |
| ≥50 | 2720 | 37.9 | 1955 | 35.9 | 765 | 44.1 |  |
| Sex |  |  |  |  |  |  | 0.136 |
| Male | 5646 | 78.7 | 4258 | 78.3 | 1388 | 80.0 |  |
| Female | 1530 | 21.3 | 1182 | 21.7 | 348 | 20.0 |  |
| Marital status |  |  |  |  |  |  | <0.001 |
| Single | 2405 | 33.5 | 1891 | 34.8 | 514 | 29.6 |  |
| Married | 3659 | 51.0 | 2723 | 50.1 | 936 | 53.9 |  |
| Divorced and others | 1112 | 15.5 | 826 | 15.2 | 286 | 16.5 |  |
| Transmission route |  |  |  |  |  |  | <0.001 |
| Heterosexual contact | 4526 | 63.0 | 3330 | 61.2 | 1196 | 68.9 |  |
| Homosexual contact | 2267 | 31.6 | 1852 | 34.0 | 415 | 23.9 |  |
| Injection drug use | 55 | 0.8 | 37 | 0.7 | 18 | 1.0 |  |
| Unknown | 328 | 4.6 | 221 | 4.1 | 107 | 6.2 |  |
| Baseline CD4 counts (cells/mm^3^) |  |  |  |  |  |  | <0.001 |
| <200 | 3598 | 50.1 | 2594 | 47.7 | 1004 | 57.8 |  |
| 200–499 | 3034 | 42.3 | 2420 | 44.5 | 614 | 35.4 |  |
| ≥500 | 428 | 6.0 | 341 | 6.3 | 87 | 5.0 |  |
| Missing | 116 | 1.6 | 85 | 1.6 | 31 | 1.8 |  |
| Last ART regimen |  |  |  |  |  |  | <0.001 |
| Recommended first-line regimen | 5678 | 79.1 | 4373 | 80.4 | 1305 | 75.2 |  |
| Alternative first-line regimens | 202 | 2.8 | 122 | 2.2 | 80 | 4.6 |  |
| Second-line regimens | 1296 | 18.1 | 945 | 17.4 | 351 | 20.2 |  |
| Duration from HIV diagnosis to ART (days) |  |  |  |  |  |  | 0.072 |
| >30 | 2664 | 37.1 | 1988 | 36.5 | 676 | 38.9 |  |
| ≤30 | 4512 | 62.9 | 3452 | 63.5 | 1060 | 61.1 |  |
| TB coinfection |  |  |  |  |  |  | <0.001 |
| No | 6464 | 90.1 | 4969 | 91.3 | 1495 | 86.1 |  |
| Yes | 679 | 9.4 | 451 | 8.3 | 228 | 13.1 |  |
| Missing | 33 | 0.5 | 20 | .4 | 13 | .7 |  |
| Hepatitis B virus coinfection |  |  |  |  |  |  | 0.107 |
| No | 5179 | 72.2 | 3956 | 72.7 | 1223 | 70.4 |  |
| Yes | 645 | 9.0 | 470 | 8.6 | 175 | 10.1 |  |
| Missing | 1352 | 18.8 | 1014 | 18.6 | 338 | 19.5 |  |

***Note.*** VL, viral load; Recommended first-line regimen: tenofovir, lamivudine, and efavirenz (TDF+3TC+EFV); Alternative first-line regimens: zidovudine, lamivudine, and nevirapine (AZT+3TC+NVP), zidovudine, lamivudine, and efavirenz (AZT+3TC+ EFV), tenofovir, lamivudine, and nevirapine (TDF+3TC+NVP); Second-line regimens: regimens including lopinavir/ritonavir (LPV/r), abacavir (ABC), or other affordable self-paying therapeutic options; TB, tuberculosis; ^*^Chi-square test.
